# Ion-Imprinted Polymer Modified with Carbon Quantum Dots as a Highly Sensitive Copper(II) Ion Probe

**DOI:** 10.3390/polym13091376

**Published:** 2021-04-23

**Authors:** Zhiming Wang, Cuo Zhou, Shunwei Wu, Chunyan Sun

**Affiliations:** College of Chemical Engineering, Qinghai University, Xining 810016, China; wzm18209780460@163.com (Z.W.); zc18797317443@163.com (C.Z.); wsw13709744989@163.com (S.W.)

**Keywords:** carbon quantum dots, fluorescence quenching, ion-imprinted polymer, copper(II) ion

## Abstract

Fluorescence analysis technology and ion imprinting technology are combined to prepare a copper ion fluorescence sensor. Carbon quantum dots (CQDs), with a quantum yield of 79%, were synthesized by a hydrothermal process using citric acid as the carbon source. The prepared CQDs, acting as the fluorophore, were grafted onto the surface of an SBA-15 mesoporous molecular sieve by an amidation reaction. Then, the fluorescent sensor CQDs@Cu-IIP was prepared using a surface imprinting technique with the modified SBA-15 as the substrate, copper ions as a template, tetraethoxysilane as the crosslinker, and 3-aminopropyl-3-ethoxysilane as the functional monomers. The sensor showed strong fluorescence from CQDs and high selectivity due to the presence of Cu(II)-IIP. After the detection conditions were optimized, the fluorescence intensity of the sensor had good linearity with Cu(II) concentration in a linear range of 0.25–2 mg/L and 3–10 mg/L. This CQDs@Cu-IIP was applied to the determination of traces Cu(II) in real water samples and good recoveries of 99.29–105.42% were obtained. The present study provides a general strategy for fabricating materials based on CQDs for selective fluorescence detection of heavy metals.

## 1. Introduction

Higher energy demand and increased use of heavy metals in industrial processes have increased human exposure to these toxic substances in drinking water [1]. Different heavy metals bring different harm to the environment and the human body. For example, copper sulfate has a stimulating effect on the gastrointestinal tract and may cause nausea, vomiting, and heartburn. In severe cases, abdominal cramps, hematemesis, and black stools occur [2,3]. Copper ions combine with many organic substances and may destroy the normal environment and physiological processes [4]. Therefore, the determination of trace-level heavy metal ions in complex solutions is of great significance [5].

Until now, many traditional detection methods, such as ICP-MS, ICP-OES, AAS and electrochemistry, have been used to detect Cu(II) levels in environmental water [6,7,8,9,10]. However, these instrumentation techniques are often complex, expensive, time-consuming, and require tedious sample pretreatment [11]. Compared with these traditional detection methods, fluorescence detection has great potential owing to its excellent properties such as high sensitivity and quick process [12]. Carbon quantum dots (CQDs), as a new type of carbon nanomaterial, have aroused extensive interest due to their unique properties, such as low cost, ease of manufacture, non-toxicity, stability. It has potential applications in many areas such as biosensing, electrocatalyst, photoscience, and so on, especially in the field of fluorescence recognition [13,14,15,16]. A large number of researches on metal ion detection based on CQD have been reported [17,18,19]. However, fluorescent recognition based on CQDs often has interferences from coexisting ions, leading to low selectivity. Therefore, a feasible method for improving the selectivity of fluorescent detection based on CQDs is the use of ion imprinting polymers (IIPs) with specific recognition sites for target ions. IIPs have been widely used in recognition for metal ions owing to their high selectivity [20]. Chen et al. reported a glass fiber paper-based analytical instrument based on QDs and copper ion-imprinted polymers. A new type of highly selective fluorescence sensor was fabricated, and on this basis, a miniature (3D) origami ion-imprinted polymer microfluidic paper-based chip device was proposed for specificity to Cu^2+^ and Hg^2+^, sensitive and multiple detections. In this device, the surface of the paper is activated by amino treatment and grafting of CdTe QDs complex, resulting in QD fluorescence quenching. This method can simultaneously detect Cu^2+^ and Hg^2+^ ions, with good selectivity and sensitivity. This simple, fast, and reliable sensing strategy opens up attractive prospects for ion monitoring in aqueous samples [11,21]. The integration of the fluorescence of CQDs and selectivity of IIPs are expected to provide a synergistic effect in sensor construction. So far, there are limited reports on the combination of ion-imprinted polymers and carbon quantum dots [22].

In this work, CQDs were fabricated by a hydrothermal treatment using citric acid as a carbon source. The prepared CQDs, acting as the fluorophore, was grafted onto the surface of an SBA-15 mesoporous molecular sieve by an amidation reaction. Then, the fluorescent sensor CQDs@Cu-IIP was prepared by a surface imprinting technique. The sensor was used to detect Cu(II) in real water samples and shows high selectivity and sensitivity.

## 2. Experimental Section

### 2.1. Materials

Triblock copolymer Pluronic P123 ((EO)20(PO)70(EO)20), 1-(3-dimethylaminopropyl)-3-ethylcarbodiimide hydrochloride (EDC) and 3-aminopropyl-3-ethoxysilane (APTES) were purchased from Aladdin Reagent Co., Ltd. (Shanghai, China). Tetraethoxysilane (TEOS), citric acid, ethylenediamine (EDA), hydrochloric acid, ammonia solution, *N*-hydroxysuccinimide (NHS), Cu_2_SO_4_, Na_2_HPO_4_, and NaH_2_PO_4_ were purchased from Sinopharm Chemical Reagent Co., Ltd.(Shanghai, China). All chemicals were of at least analytical grade. Ultrapure water was used during the entire experimental process. River water was collected from the Beichuan river in Xining.

### 2.2. Synthesis of CQDs

The CQDs were synthesized in one step by the classical hydrothermal method using citric acid as the carbon source. First, 1.0 g of citric acid and 335 μL of EDA were dissolved in 10 mL of ultrapure water. The mixed solution was sealed in a 25 mL Teflon-lined stainless steel vessel and heated at a rate of 2 °C/min to a holding temperature of 150 °C for 5 h. The CQDs solution was first filtered through a 0.22 μm membrane to remove macromolecular impurities. The resulting solution was dialyzed for 24 h to remove any unreacted precursors. After completion of the dialysis, the solution was placed in a Petri dish to freeze-dry, and finally, a dark-yellow powder solid was obtained.

### 2.3. Preparation of Amino-Functionalized SBA-15

The SBA-15 molecular sieves were synthesized using a classical preparation method [23], not described here. 1.0 g of SBA-15 and 100 mL of 3 mol/L hydrochloric acids were added into a 250 mL flask and refluxed at 75 °C for 24 h. The solid was washed until the filtration was neutral. The filtered solid was dried at 60 °C for 6 h to obtain activated SBA-15. Then, 1.0 g of activated SBA-15 and 20 mL of APTES were added into 80 mL of water and stirred at 50 °C for 4 h under a nitrogen atmosphere. The solid was separated, washed with water, and dried at 60 °C for 6 h to obtain amino-functionalized SBA-15 (SBA-15-NH_2_).

### 2.4. Synthesis of SBA-15-CQDs

SBA-15-CQDs were synthesized using an amide condensation reaction between the -NH_2_ groups on the surface of SBA-15 and the −COOH groups on the CQDs. Specifically, 0.10 g of CQDs powder was dissolved in 10 mL of water. Then, 10 mL of 20 mg/mL EDC solution and 10 mg/mL of NHS solution were added and sonicated for 10 min. Then, 1.0 g of SBA-15-NH_2_ was added to the mixed solution, and magnetically stirred at room temperature for 18 h. After that, a pale-yellow solid powder of SBA-15-CQDs was obtained.

### 2.5. Synthesis of CQDs@Cu-IIP

First, 0.500 g of SBA-15-CQDs and 0.005 g of copper sulfate were mixed in 20 mL of water for 5 min. Then, 76 μL of APTES was added, and it was stirred for 30 min. Next, 100 μL of TEOS and 250 μL of concentrated ammonia water were sequentially added to the mixed solution, followed by stirring for 4 h at room temperature. The product was collected by centrifugation. The Cu(II) templates in the IIP were extracted with 0.1 mol/L of hydrochloric acid until no Cu(II) could be detected by FAAS in the eluent. Finally, the CQDs@Cu-IIP were dried at 40 °C under vacuum for 12 h. A non-imprinted polymer sensor (CQDs@NIP) was prepared as a blank, without the addition of copper sulfate.

### 2.6. Material Characterization

X-ray powder diffraction (XRD) patterns were obtained on an X-ray diffractometer (D8 FOCUS, Bruker, Karlsruhe, Germany) with Cu Kα radiation. The chemical composition of materials was acquired with an X-ray photoelectron spectrometer (ESCALAB Xi+, Thermo Fisher Scientific, Waltham, MA, USA) using a monochromatic Al Kα source (1486.6 eV). The infrared spectra in KBr were obtained using an FT-IR BXII spectrometer (Perkin-Elmer, Waltham, MA, USA). The surface charge of CQDs was measured using a Zeta potential analyzer (Zetasizernano, Malvern Instruments, Shanghai, China). The morphologies were observed by scanning electron microscope (JSM-6610LV, JEOL., Ltd., Akishima City, Tokyo, Japan) and transmission electron microscope (JEM-2100F JEOL., Ltd., Akishima City, Tokyo, Japan). The fluorescence experiment was performed using an FL-7000 spectrofluorometer (FL-7000, Hitachi, Tokyo, Japan). Perform liquid and solid absorption studies of UV-Vis samples on a spectrophotometer (T6, general analysis, China and Cary Series UV-Vis-NIR, Agilent Technologies, Beijing, China). Detection of metal ions using flame atomic absorption spectrophotometer (AAS, A-6300C, Shimadzu, Kyoto, Japan). The sample was dried using a vacuum freeze dryer (SCIENTZ-12N, Ningbo Xinzhi Biological Technology Co., Ltd., Shanghai, China).

### 2.7. Determination of the Fluorescence Quantum Yield

Fluorescence measurements were performed under the following conditions: the slit widths of the emission and excitation gap widths were 2.5 and 5 nm, respectively, and the excitation wavelength was set at 366 nm. A scan rate of 240 nm/min, and a photomultiplier tube voltage of 400 V were used. The change in fluorescence intensity of the corresponding emission wavelength was used to determine the optimal excitation wavelength.

The CQDs quantum yield (QY) was determined via a comparative method using quinine sulfate (QY = 54.8%) as the reference solution with an excitation wavelength at 366 nm [24]. The absorption value at 366 nm excitation wavelength was maintained below 0.1 to minimize self-absorption. Quinine sulfate was dissolved in 0.1 mol/L H_2_SO_4_, and the CQDs were dissolved in distilled water. The QY of CQDs was calculated using the following equation:

(1) $$\varphi_{S}=\varphi_{R}\frac{F_{S}}{F_{R}}\frac{A_{R}}{A_{S}}\frac{\eta_{S}{{}^{2}}_{{}_{}}}{\eta_{R}{}^{2}}$$

where *φ* stands for the fluorescence quantum yield, *F* refers to the integrated fluorescence intensity, *η* is the refractive index, and *A* is the absorption value which is measured on a UV-Vis spectrophotometer. The subscripts “*R*” and “*S*” correspond to the reference and the sample, respectively.

## 3. Results and Discussion

### 3.1. Synthesis of CQDs and CQDs@Cu-IIP

**Figure 1:**
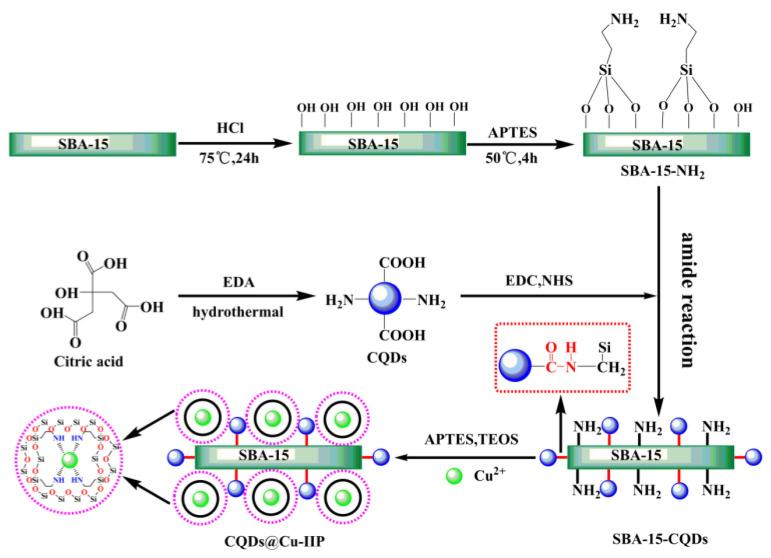
The synthetic route of CQDs@Cu-IIP. Physical and optical properties of CQDs and CQDs@Cu-IIP.

The synthetic route of CQDs@Cu-IIP is shown in Figure 1. First, the prepared SBA-15 was activated and aminated to obtain SBA-15-OH and SBA-15-NH_2_. Second, the CQDs were prepared by citric acid (CA) and ethylene-diamine (EDA) through an improved hydrothermal method. The CQDs were grafted onto the surface of SBA-15-NH_2_ through an amide reaction. The remaining amino groups in SBA-15-NH_2_ were used to synthesize ion-imprinted polymers by surface imprinting technology. In this study, the surface imprint strategy was used because it controls the imprint location to be near the surface or on the surface of the material, thereby improving accessibility and reducing response time [25].

### 3.2. Physical and Optical Properties of CQDs and CQDs@Cu-IIP

**Figure 2:**
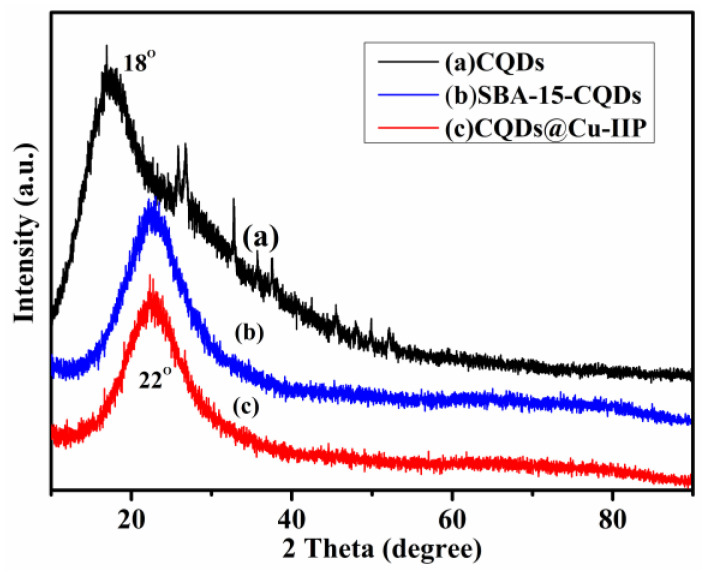
XRD patterns of CQDs (**a**), SBA-15-CQDs (**b**) and CQDs@Cu-IIP (**c**).

Figure 2 shows the X-ray diffraction patterns of the CQDs, SBA-15-CQDs and CQDs@Cu-IIP. For the CQDs, when the 2θ angle is about 18° and 22°, a distinct broad peak appears, which is related to highly disordered carbon atoms [26]. The peaks correspond to the interlayer spacing d is 0.49 nm, which is slightly longer than the spacing between the (002) planes in bulk graphite (0.34 nm). The increase of the d value indicates the increase of the amorphous nature, attributed to the introduction of more defect sites into the product by increasing the *N* doping of the crystal lattice [27,28].

**Figure 3:**
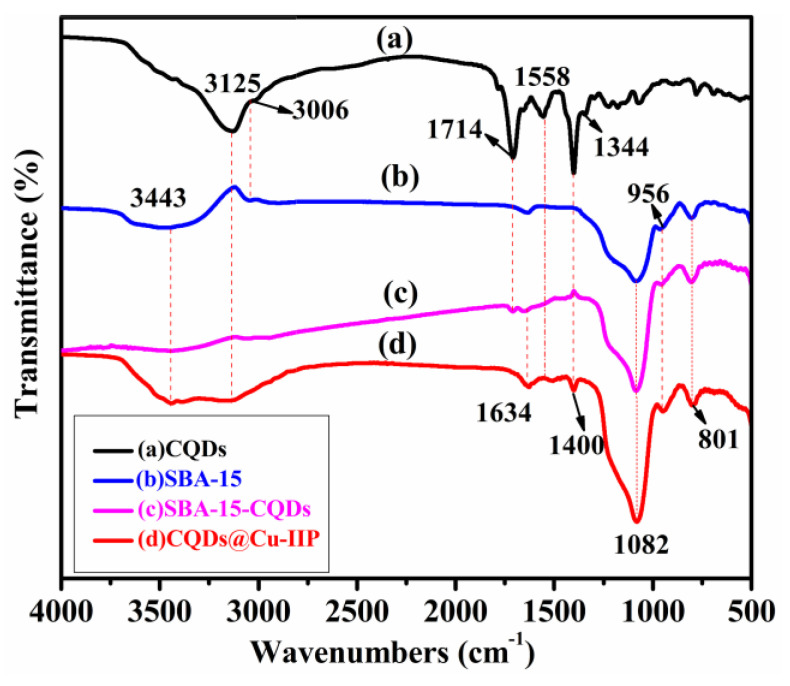
The FT-IR spectra of CQDs (**a**), SBA-15 (**b**), SBA-15-CQDs (**c**) and CQDs@Cu-IIP (**d**).

FT-IR spectra of the materials are shown in Figure 3. For CQDs (Figure 3a), the absorption peak at 3125 cm^−1^ represents the stretching vibration of O–H bonds. The peaks at 1714 cm^−1^ are ascribed to the stretching vibration of C=O [29]. The characteristic absorption bands of C–N and N–H were also observed at 1400 cm^−1^ and 3006 cm^−1^, respectively. The absorption peaks at 1558 cm^−1^ and 1344 cm^−1^ are due to C=C stretching vibration and C–H in-plane bending vibration, respectively [30]. It can be concluded that the as-synthesized CQDs have numerous hydrophilic groups, such as hydroxyl, carboxylic, and amino moieties [31]. These groups are helpful in enhancing the aqueous solubility of CQDs for further modification. In the spectrum of the synthesized SBA-15 (Figure 3b), asymmetric stretching and symmetrical stretching of Si–O–Si is observed at 1082 cm^−1^ and 801 cm^−1^, respectively, and bending vibration of Si–OH and a symmetrical tensile vibration of Si–O–Si are observed at 956 cm^−1^ and 801 cm^−1^, respectively [32]. The absorption peak of 1634 cm^−1^ for SBA-15-CQDs and CQDs@IIP (Figure 3c,d) is caused by the asymmetric C=O vibrations of the amide groups [33].

**Figure 4:**
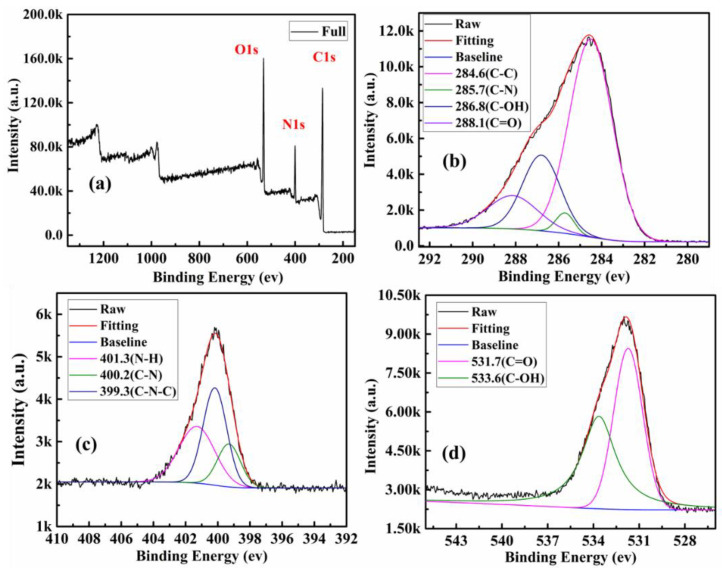
XPS analysis of as-prepared CQDs, (**a**) XPS survey spectrum of the CQDs; (**b**) high-resolution spectrum of C1s; (**c**) high-resolution spectrum of O1s; and (**d**) high-resolution spectrum of N1s.

**Figure 5:**
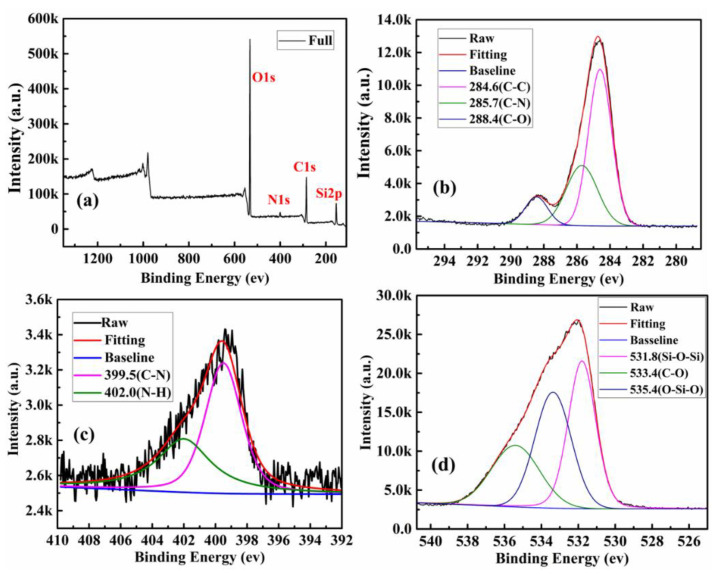
XPS analysis of as-prepared SBA-15-NH_2_, (**a**) XPS survey spectrum of the SBA-15-NH_2_; (**b**) high-resolution spectrum of C1s; (**c**) high-resolution spectrum of N1s; and (**d**) high-resolution spectrum of O1s.

**Figure 6:**
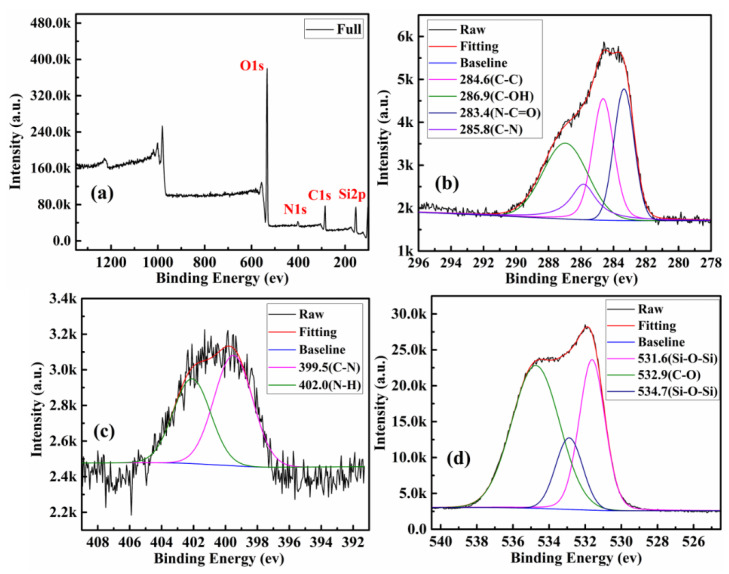
XPS analysis of as-prepared SBA-15-CQDs, (**a**) XPS survey spectrum of the SBA-15-CQDs; (**b**) high-resolution spectrum of C1s; (**c**) high-resolution spectrum of N1s; and (**d**) high-resolution spectrum of O1s.

**Figure 7:**
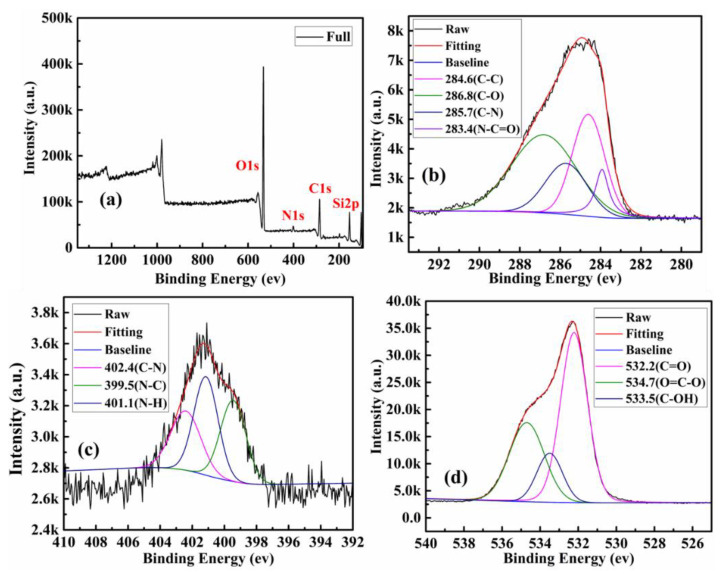
XPS analysis of as-prepared CQDs@IIP, (**a**) XPS survey spectrum of the CQDs@IIP; (**b**) high-resolution spectrum of C1s; (**c**) high-resolution spectrum of N1s; and (**d**) high-resolution spectrum of O1s.

XPS was used to ascertain the element analysis and functional groups present on the surface. The chemical compositions of the surface of the product of all steps are shown in Table 1. Among them, SBA-15-NH_2_ contains 4.18% nitrogen, indicating that SBA-15 was successfully aminated. The XPS survey spectrum further illustrates the surface chemical composition of CQDs (Figure 4a). CQDs displayed three predominant strong peaks at 284.6, 399.9, and 532.4 eV, which were ascribed to C1s, N1s and O1s peak, respectively. The contents of carbon, nitrogen and oxygen were calculated to be 67.77%, 11.94% and 20.29%, respectively. As shown in Figure 4b, the C1s spectrum shows five peaks at 284.6, 285.7, 286.8 and 288.1eV, which were assigned to C–C/C=C, C–N, C–O and C=O groups, respectively [34]. The high-resolution spectrum of N1s shows four peaks at 399.3, 400.2 and 401.3 eV (Figure 4c), which were attributed to C–N–C, C–N and N–H groups, respectively [35]. From the O1s spectrum shown in Figure 4d, the two fitted peaks at 531.7 and 533.6 eV were ascribed to C=O and C–OH groups, severally [36,37]. The same analysis was performed on SBA-15-NH_2_, SBA-15-CQDs, and CQDs@IIP. The surface functional groups and elemental analysis results were shown in Figure 5, Figure 6 and Figure 7. FT-IR and XPS results confirmed that the surface of SBA-15 was successfully modified with APTES, and the imprinted polymer was successfully synthesized.

**Figure 8:**
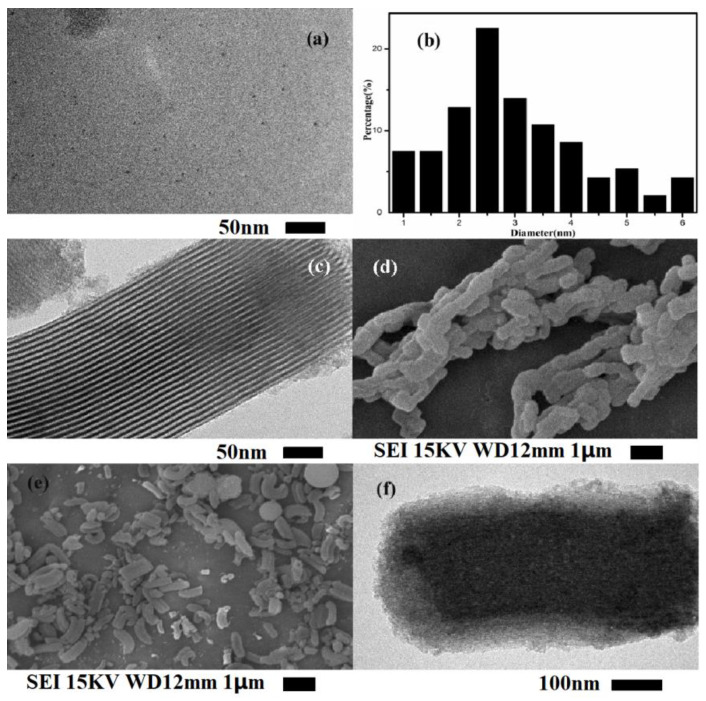
TEM image of CQDs (**a**) and particle size statistics of CQDs (**b**); TEM image of SBA-15 (**c**); SEM image of SBA-15-CQDs (**d**); SEM (**e**) and TEM (**f**) image of CQDs@Cu-IIP.

The morphology of the materials was examined by SEM and TEM. Figure 8a,b shows that the CQDs were highly dispersive and microspherical particles in the range of 4–8 nm in size. As seen in Figure 8c image, transmission electron micrographs of SBA-15 molecular sieves can clearly observe the long-range ordered structure of mesoporous substrates. The diameter of the pores is about 9 nm, which is consistent with the literature [38,39]. Figure 8d shows SBA-15-CQD of SEM image, the material has a rod-like morphology and a certain degree of agglomeration, which may be caused by the amidation reaction of carbon quantum dots with two or more SBA-15 surface groups. The SEM image of CQDs@Cu-IIP is shown in Figure 8e, and the rod-like morphology of the SBA-15 is maintained, indicating that the modification and imprinting processes occur on the surface of the base material without causing deformation of the base structure. The pore structure of SBA-15 is difficult to observe in Figure 8f, indicating that the pores have been covered or are occupied by the CQDs. From the TEM image of the CQDs@Cu−IIP, the IIP shell (the lighter edge region) is clearly visible, and the approximate thickness of the IIP coating is calculated to be 50 nm.

**Figure 9:**
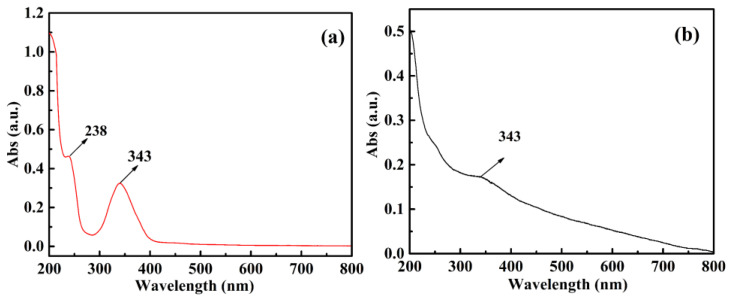
UV-Vis spectra of (**a**) CQDs (0.05 g/L) and (**b**) CQDs@Cu-IIP (0.05 g/L) in aqueous solution.

To explore the optical properties of the materials, UV-Vis absorption and FL spectra were studied at room temperature. The UV–Vis absorption spectrum of CQDs and CQDs@Cu−IIP were shown in Figure 9. For CQDs, the sharp absorption peak at 238 nm is assigned to the π–π* transition of C=C bonds in the aromatic ring, and the peak at 343 nm is assigned to the n–π* transition of C=O bonds [29]. Compared with the bare CQDs, the absorbance of CQDs@Cu-IIP at 343 nm is lower that may be due to the reduction of carboxyl groups on the surface of CQDs after the polymerization process.

**Figure 10:**
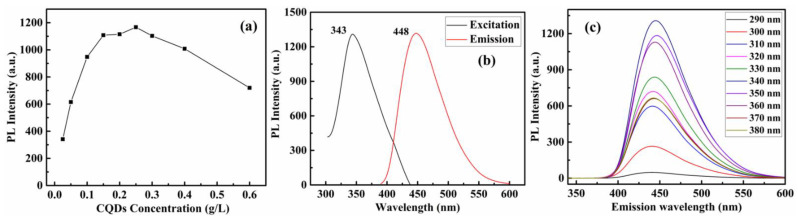
(**a**) The fluorescence intensity of CQDs at different concentrations in the aqueous solution. (**b**) Fluorescence excitation and emission spectra of 0.25 g/L CQDs. (**c**) Emission spectra of 0.25 g/L CQDs at different excitation wavelengths.

The fluorescence intensity of CQDs with different concentrations is shown in Figure 10a. It is found that when the concentration of CQDs is greater than 0.25 g/L, the fluorescence of CQDs will be decline which is caused by the inner filter effect. The 0.25 g/L CQDs is used in the subsequent fluorescence detection, where the fluorescence intensity is close to the maximum. Figure 10b shows the fluorescence excitation and emission spectra of CQDs aqueous solution. From the excitation spectrum, it can be seen that the maximum fluorescence excitation wavelength of CQDs is 343 nm which is consistent with the result of UV–vis absorption spectrum. When excited at 343 nm, the maximum fluorescence emission wavelength of CQD is 448 nm. Thus, an excitation wavelength at 343 nm was used in further experiments. To further investigate the optical properties, FL emission spectra of the CQDs were recorded at various excitation wavelengths. As shown in Figure 10c, the FL spectra presented a representative excitation wavelength-independent characteristic. The emission wavelength of CQDs does not red shift or blue shift with the change of excitation wavelength. This may be related to the uniform size of the prepared CQDs. Under the irradiation of 365 nm ultraviolet light, the CQDs solution turned bright blue, and the quantum yield (QY) of CQDs was calculated to be about 79%.

### 3.3. CQDs@Cu-IIP Sensing Properties 

**Figure 11:**
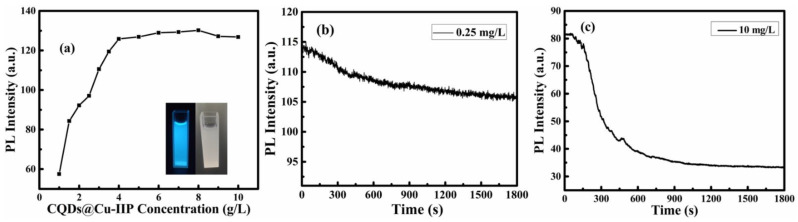
(**a**) The fluorescence intensity of CQDs@Cu-IIP at different concentrations in the aqueous solution. Inset shows the images of 4 g/L CQDs@Cu-IIP aqueous solutions under 365 nm ultraviolet lamp and daylight lamp. (**b**) Fluorescence–time curve of 0.25 mg/L copper ion solution containing 4 g/L CQDs@Cu-IIP. (**c**) Fluorescence–time curve of 10 mg/L copper ion solution containing 4 g/L CQDs@Cu-IIP. (*E*_x_ = 343 nm).

The effects of different reactive conditions (the concentration, reactive time and pH) on the detection of Cu^2+^ were investigated to achieve excellent sensitivity. The concentration of the ion-imprinted polymer in the detection system plays an important role in the detection range and sensitivity. The effect of concentration is illustrated in Figure 11a. When the concentration reaches 4 g/L, the intensity plateaued and did not significantly increase beyond 4 g/L. Therefore, in the next experiment, the concentration of CQDs@Cu−IIP was set at 4 g/L.

Reaction time is also an important factor in fluorescence detection. We study the effect of contact time on fluorescence intensity at two different copper ion concentration conditions (10 mg/L and 0.25 mg/L). As shown in Figure 11b,c, the fluorescence intensity of both solutions decreased with the increase of time, and the fluorescence intensity stabilized at 13–15 min. This is consistent with the adsorption equilibrium time of most reported ion-imprinted polymers [40]. Reliably, this indicates that the fluorescent material has application prospects in the rapid detection of copper ions. Therefore, in the following experiments, the reaction time of CQDs@Cu-IIP with Cu^2+^ ions is controlled to 15 min. 

**Figure 12:**
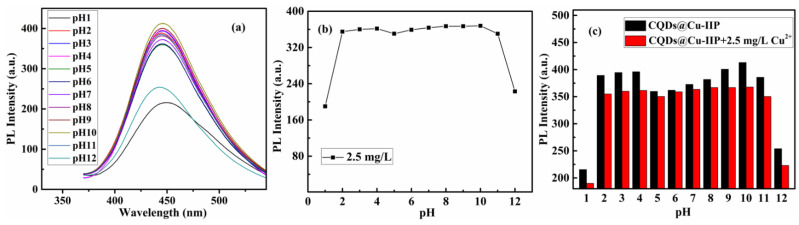
(**a**) The relationship between the pH values and the fluorescence intensity of 4 g/L CQDs@Cu-IIP solutions. (**b**) The relationship between the pH values and the fluorescence intensity of 4 g/L CQDs@Cu-IIP containing 2.5 mg/L copper ions. (**c**) The comparison results are shown as a column chart. (*E*_x_ = 343 nm, the pH was adjusted by 1 mol/L sodium hydroxide solution).

The pH of an aqueous solution is one of the important parameters affecting molecular affinity. We studied the fluorescence intensity of CQDs@Cu-IIP at different pH levels ranging from 1 to 12 to determine the optimum pH during the assay. The effect of the pH of the reactive solution is depicted in Figure 12a. The fluorescence intensity of CQDs@Cu-IIP under alkaline conditions is higher than under acidic conditions. In addition, when the pH of the solution is less than 3 or higher than 10, the fluorescence is low, and when the pH is 10, the fluorescence intensity of the solution reaches a maximum. Due to the low concentration, it will not affect the measurement of copper ions when the pH of the solution is 10. Moreover, the influence mechanism of the pH was further studied. In acidic conditions, CQDs agglomerate under the action of hydrogen bonds and protonation, causing fluorescence quenching of the CQDs. In addition, as there are many carboxyl and hydroxyl groups on the surface of CQDs, when the pH of the aqueous solution increases, the H^+^ of the CQDs combines with OH– and becomes H_2_O. In addition, the results of the probe’s sensing performance for copper ions at different pH values are shown in Figure 12b. In the range of pH 3–10, the probe’s responsiveness to copper ions can maintain excellent stability. The results show that the probe can achieve quantitative detection of copper ions in a wide pH range. To clearly identify the optimum pH, the results of the comparison between Figure 12a,b have been shown as a column chart in Figure 12c. It can be seen that the probe exhibits maximum fluorescence quenching when the pH is 10.

**Figure 13:**
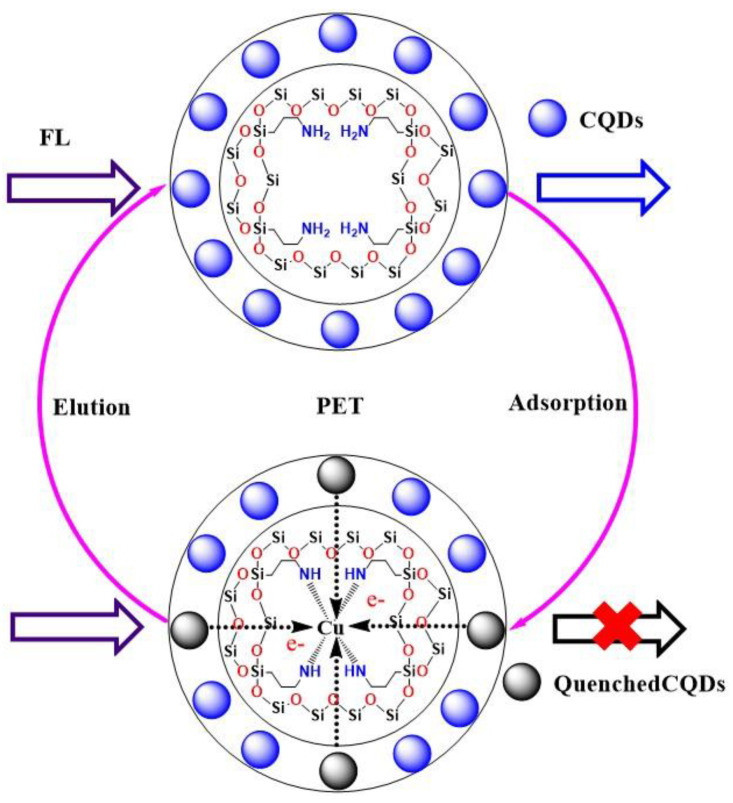
Fluorescence quenching process: Photo-induced electron transfer.

Under the current research conditions, the capability of the CQDs@Cu-IIP for the quantitative determination of Cu^2+^ was further studied based on fluorescence quenching due to Cu(II). During the sensing process, copper ions were strongly absorbed by CQDs@Cu-IIP due to specific recognition sites and coordination bond interactions between copper ions and amino groups [23]. The zeta potential of CQDs dispersed in the aqueous solution is −7.39 mV. When CQDs carrying negative charges are excited, copper ions act as electron acceptors and cause the photo-induced Electron Transfer (PET) effect of CQDs. So as shown in Figure 13, the CQDs are difficult to excite, causing a fluorescence quenching phenomenon.

### 3.4. Fluorescence Detection of Cu(II) ion by CQDs@Cu-IIP

**Figure 14:**
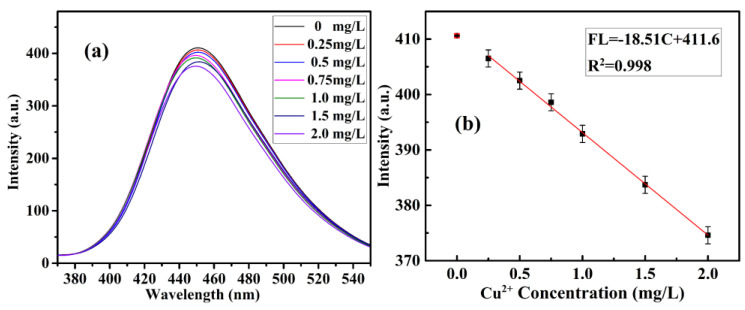
Fluorescence spectra (**a**) and linear fitting equations (**b**) of CQDs@Cu−IIP (4 g/L) and different concentrations of Cu^2+^, 0.25–2 mg/L. (*E*_x_ = 343 nm, pH = 10).

**Figure 15:**
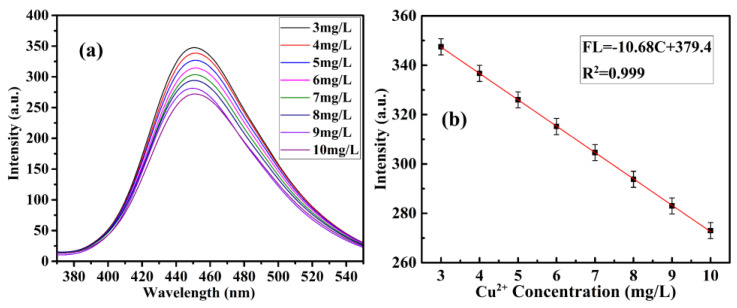
Fluorescence spectra (**a**) and linear fitting equations (**b**) of CQDs@Cu−IIP (4 g/L) and different concentrations of Cu^2+^, 3–10 mg/L. (*E*_x_ = 343 nm, pH = 10).

To realize the ion detection function of CQDs@Cu-IIP, they were added to a series of different concentrations of copper ion solutions to detect the fluorescence quenching condition based on the change of fluorescence intensity after the addition. The fluorescence intensity versus the Cu^2+^ concentration was plotted. Using this curve, the Cu^2+^ concentration of an unknown solution can be detected, and the detection limit of copper ions by CQDs@Cu-IIP can be calculated. The fluorescence of CQDs@Cu-IIP was effectively quenched by Cu^2+^ ions at different concentrations, as shown in Figure 14 and Figure 15. The quenching efficiencies were fitted by the following two linear equations, in which the equation of FL = −18.51C + 411.6 (R^2^ = 0.998) was suitable for low-level determination (0.25–2 mg/L), whereas FL = −10.68C + 379.4 (R^2^ = 0.999) was suitable for high level determination (3–10 mg/L). The detection limits of Cu^2+^ ions were estimated to be 0.016 and 0.028 mg/L. The two fitting slopes represent the different quenching speeds of the probe at different copper ion concentrations.

In addition, we compared the properties of our probe with some previously reported sensors, and the results were listed in Table 2. By combining CQDs and Cu-IIP, the sensing performance of the probe can be improved, and the most prominent is that the detection range of copper ions is greatly improved.

**Figure 16:**
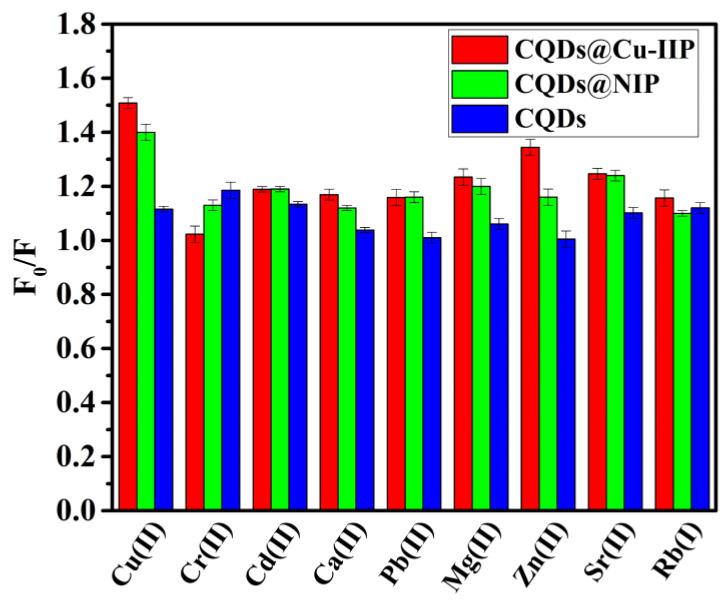
Selectivity of CQDs@Cu-IIP(4 g/L), CQDs@NIP(4 g/L) and CQDs(0.3 g/L). (*E*_x_ = 343 nm, pH = 10).

To study the CQDs@Cu-IIP fluorescence sensor selectivity, the influence of common interference ions such as Cr^2+^, Cd^2+^, Ca^2+^, Pb^2+^, Mg^2+^, Zn^2+^, Sr^2+^, and Rb^+^ (all at 10 mg/L) in the presence of 4 g/L CQDs@Cu-IIP were investigated. The results in the presence of 0.3 g/L CQDs and 4 g/L CQDs@NIP were also recorded for comparison. It can be clearly seen from Figure 16 that CQDs@Cu-IIP has a higher quenching ratio for copper ions over other metal ions. In addition, the selectivity of CQDs@Cu-IIP for Cu^2+^ ions is much better than that of CQDs@NIP and CQDs. This can be attributed to that CQDs@Cu-IIP have cavities that are complementary in shape and functionality to the template Cu^2+^ ions.

**Figure 17:**
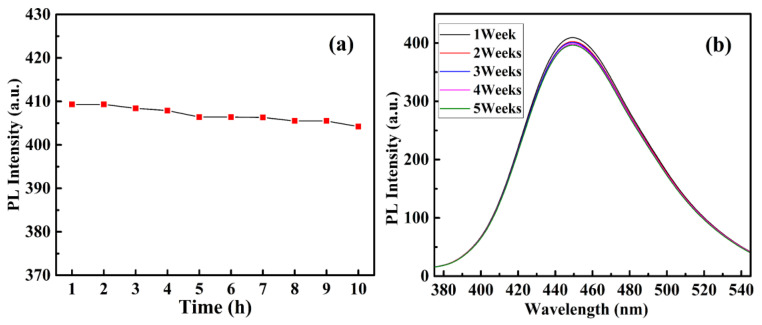
Light-stability (**a**) and storage stability (**b**) of 4 g/L CQDs@Cu-IIP. (*E*_x_ = 343 nm).

As the important influence factors, the light-stability and storage-stability of CQDs@Cu-IIP were also studied. Using a tungsten lamp to continuously irradiate 4 g/L CQDs@Cu-IIP solutions for 10 h, and measures the fluorescence intensity of the sample every hour, the results are shown in Figure 17a. The fluorescence intensity of the sensor is almost unchanged after 3 h, but it decreases by 1.3% after 10 h. If the solution is stored in the dark, the fluorescence intensity is almost constant within one week, but decreases by 3.6% after 5 weeks, as shown in Figure 17b. Therefore, 3 h is chosen as the effective photoinduction time of CQDs@Cu-IIP, and the sensor can be used to quantitatively detect copper ions within one week.

### 3.5. CQDs@Cu-IIP for Analysis of Tap Water Samples

To further verify the practical utility of the prepared CQDs@Cu-IIP, it was used for the detection of copper ions in tap water and Beichuan River water. All real samples were firstly filtered through a 0.45 μm polycarbonate membrane, and the pH values were adjusted to 10 using the 1 mol/L NaOH. The CQDs@Cu-IIP (4 g/L) was used as a probe, at an excitation wavelength of 343 nm, the concentration of copper ions in two actual water samples was tested by fluorescence analysis, and the spike recovery rates were calculated. The detection results were compared with that of the atomic absorption spectrometer (AAS) to discuss the feasibility of CQDs@Cu-IIP sensor. As shown in Table 3, the recovery rates obtained from fluorescence spectrometry were between 99.29% and 105.42%, which was no significant difference compared with the AAS method. It shows that the fluorescence sensor has certain practical applications.

## 4. Conclusions

Combining the advantages of ion imprinting polymers and the unique fluorescent properties of carbon quantum dots, a metal ion fluorescence sensor with the ion recognition method was successfully synthesized. The fluorescence intensity at an excitation wavelength of 343 nm was significantly quenched upon the addition of copper(II) ions, and it could be used to quantitatively detect copper(II) ions over a wide concentration range. Satisfactory results demonstrated that the CQDs@Cu-IIP could be used as a sensor to detect trace copper(II) ions in solutions.

## Figures and Tables

**Table 1:** Chemical composition obtained by XPS for products.

Components	CQDs		SBA-15-NH_2_		SBA-15-CQDs		CQDs@IIP	
	BE(eV)	Atomic%	BE(eV)	Atomic%	BE(eV)	Atomic%	BE(eV)	Atomic%
C	285.93	67.77	285.3	34.86	285.73	20.22	285.71	30.40
N	400.28	11.94	400.45	4.18	401.16	2.85	401.59	3.42
O	531.92	20.29	532.84	43.31	533.55	53.89	532.95	46.46
Si	-	-	103.81	17.65	103.37	20.04	102.97	19.72

**Table 2:** Comparison of the proposed probe with various reported sensors.

Probe	Detection Limit	Detection Range	Reference
Ce(III)/Tb(III)-doped SrF_2_	0.11–0.14 µM	0–70.0 µM	[2]
Carbon dots Sensor	0.10µM	0.7–4.0 µM	[41]
double-stranded DNA	0.29µM	0.5–10 µM	[42]
Carbon dots Sensor	23 nM	0–10 µM	[43]
B, N-CQDs	0.52 nM	0.5–10, 10–2000 nM	[44]
Carbon dots Sensor	16.84 µM	0–1870 µM	[45]
CQDs@Cu-IIP	2.86 µM (0.0016 mg/L)	3.93–31.47, 47.21–157.37 µM (0.25–2, 3–10 mg/L)	This work

**Table 3:** Determination of copper in real water samples.

Sample	Spike (mg/L)	CQDs@Cu-IIP Found (mg/L)	Recovery ^a^ (%)	AAS Found (mg/L)	Recovery ^a^ (%)
Tap Water	0	0.6468	-	0.6421	-
	1	1.6397	99.29	1.6303	98.82
	5	5.8278	103.62	5.6901	100.96
Beichuan River Water	0	0.4592	-	0.4250	-
	1	1.4766	101.74	1.4060	98.10
	5	5.7301	105.42	5.4970	101.44

^a^ Mean ± standard deviation. (*n* = 7).

## Data Availability

The data presented in this study are available on request from the corresponding author.
